# Error in the Affiliation

**DOI:** 10.1001/jamanetworkopen.2025.21937

**Published:** 2025-06-17

In the Invited Commentary titled “Combination Therapy in Metastatic Hormone-Sensitive Prostate Cancer—Are We Moving Fast Enough?”^1^ published online on May 8, 2025, there was a typographical error in the affiliation. This article has been corrected.

## References

[1] Barata PC, Garcia JA. Combination therapy in metastatic hormone-sensitive prostate cancer—are we moving fast enough? JAMA Netw Open. 2025;8(5):e259442. doi:10.1001/jamanetworkopen.2025.9442 40338551

